# NOA1, a Novel ClpXP Substrate, Takes an Unexpected Nuclear Detour Prior to Mitochondrial Import

**DOI:** 10.1371/journal.pone.0103141

**Published:** 2014-07-29

**Authors:** Natalie Al-Furoukh, Julia R. Kardon, Marcus Krüger, Marten Szibor, Tania A. Baker, Thomas Braun

**Affiliations:** 1 Department of Cardiac Development and Remodeling, Max-Planck-Institute for Heart and Lung Research, Bad Nauheim, Germany; 2 Department of Biology, Massachusetts Institute of Technology, Cambridge, Boston, Massachusetts, United States of America; Cornell University, United States of America

## Abstract

The mitochondrial matrix GTPase NOA1 is a nuclear encoded protein, essential for mitochondrial protein synthesis, oxidative phosphorylation and ATP production. Here, we demonstrate that newly translated NOA1 protein is imported into the nucleus, where it localizes to the nucleolus and interacts with UBF1 before nuclear export and import into mitochondria. Mutation of the nuclear localization signal (NLS) prevented both nuclear and mitochondrial import while deletion of the N-terminal mitochondrial targeting sequence (MTS) or the C-terminal RNA binding domain of NOA1 impaired mitochondrial import. Absence of the MTS resulted in accumulation of NOA1 in the nucleus and increased caspase-dependent apoptosis. We also found that export of NOA1 from the nucleus requires a leptomycin-B sensitive, Crm1-dependent nuclear export signal (NES). Finally, we show that NOA1 is a new substrate of the mitochondrial matrix protease complex ClpXP. Our results uncovered an unexpected, mandatory detour of NOA1 through the nucleolus before uptake into mitochondria. We propose that nucleo-mitochondrial translocation of proteins is more widespread than previously anticipated providing additional means to control protein bioavailability as well as cellular communication between both compartments.

## Introduction

Mitochondrial function is crucial for energy production and cellular survival. Maintain and adaption of mitochondrial functions to changing cellular needs is accomplished by complex signaling networks regulating mitochondrial effector proteins on the transcriptional or post-translational level. The majority of mitochondrial proteins is encoded in the nucleus and post-translationally imported into the mitochondrial membranes or into the matrix. Typical mitochondrial matrix proteins contain a N-terminal signaling peptide.

The NOA1 protein is a nuclear encoded mitochondrial matrix GTPase that stimulated OXPHOS activity [1]–[3]_ENREF_2_ENREF_5. Knockout studies revealed that NOA1 regulates mitochondrial protein biosynthesis by influencing mitochondrial ribosome biogenesis [4], which was also confirmed by studies on the bacterial NOA1 homolog YqeH [5], [6]_ENREF_7. Most likely, NOA1 is involved in additional cellular processes as indicated by recent reports indicating binding and potentially transport of G-quadruplex RNA or protection of cells from staurosporine induced apoptosis following knockdown [7]. Although the full range of biological activities of NOA1 still needs to be uncovered, it is clear that NOA1 is an essential mitochondrial matrix GTPase that acts as a limiting factor for mitochondrial protein synthesis and ATP production by oxidative phosphorylation.

Previous work on NOA1's function mostly concentrated on the mitochondrial localization and function. However, the NOA1 amino acid sequence contains a classical bipartite nuclear localization signal (NLS) suggesting that NOA1 also localizes to the nucleus. In fact, other proteins have been described that are localized in different cellular compartments. Such proteins might be produced as distinct isoforms, carry ambiguous targeting signals (e.g. for chloroplast and mitochondria) or contain two competing targeting signals [8], [9]. The activity of competing targeting signals most often depends on accessibility of signals, which might be regulated by ligand binding, protein-protein interactions [10] or posttranslational modifications affecting receptor interactions [11], [12]. In eukaryotes, several proteins are targeted both to chloroplast and mitochondria (in plants), to organelles and cytosol or to mitochondria and the ER. Examples of nucleo-mitochondrial proteins are less abundant. LRPPRC [13], [14], a pentatricopeptide repeat containing protein involved in the pathogenesis of human diseases [15], [16], is located in the nucleus and mitochondria. In the nucleus, LRPPRC is part of a ribonucleoprotein complex responsible for the nuclear export of mRNAs [17]. In mitochondria, LRPPRC is required for polyadenylation and translation of mitochondrial mRNAs [18]. The ELAC2 protein, responsible for RNaseZ activity, is also found in both mitochondria and the nucleus [19]. Furthermore, TERT, the catalytic subunit of telomerase, is imported into mitochondria during oxidative stress to protect the mitochondrial genome [20]. Finally, p53 has been shown to localize to mitochondria during cellular stress [21].

Here, we studied potential cellular transport routes of NOA1, which led to the discovery of a mandatory nuclear shuttling pathway relying on specific nuclear import and export signals prior to transport into mitochondria. Our results revealed that nuclear accumulation of NOA1 activates apoptosis indicating the need for tight control of NOA1 protein concentrations. To gain further insights into the pathways controlling NOA1 levels we searched for enzymes degrading NOA1 and found that NOA1 is a new substrate of matrix protease ClpXP. We reason that the initial localization of NOA1 in the nucleus is essential for its mitochondrial function and propose that NOA1 is the founding member of a new class of nucleo-mitochondrial proteins.

## Material and Methods

### Molecular Cloning

The NOA1 coding sequence (NM_019836) was amplified by PCR using proofreading high-fidelity Phusion Polymerase (Finnzymes). Two enzymatic restriction sites (5′-GGA TCC BamH1, 3′-CTC GAG SlaI) and a Flag tag (5′-GAT TAT AAG GAT GAT GAT GAT AAG-3′) were introduced by primer design. Truncation variants of NOA1 and the NOA1 NLS-mutant were produced by PCR using appropriate primer combinations. The ClpX coding sequence (NM_011802) was amplified from cDNA derived from C57/Bl6 mouse heart using the following primers: ClpX forward 5′-GGA TCC ATG TCC AGT TGC GGC GCT TGT-3′, ClpX stop reverse 5′-CTC GAG TTA GCT GTT TGC AGC ATC CGC TTG AC-3′. A detailed description of the cloning procedure is given in the supplementary materials.

### Cell culture

Mouse myoblast C2C12, fibroblast NIH 3T3, macrophage RAW264.7 cells and HEK293 cells were cultured at 37°C in humidified incubators with 8% CO_2_. All cells were grown in DMEM (4.5 g glucose, PAA) supplemented with 10% FCS, 1% penicillin/streptomycin and 1% L-glutamine. Cells were treated with Flunarizine (100 µM), leptomycin-B (5–20 ng/ml), Cycloheximide (10 ng/ml) or DMSO for the indicated times. Chemicals were purchased from Sigma Aldrich. Fractionation was performed with the Proteome Fractionation Kit (Calbiochem) following the manufacturer's protocol.

### Annexin V and propidium iodide (PI) staining for Flow cytometry

Phosphatidylserine on the cell surface was detected with Annexin V-FITC (Sigma). DNA was detected with propidium iodide (PI), (Sigma). Cells were transfected 8 hours prior staining with plasmids encoding NOA1 (wild type and ΔMTS mutant), incubated with or without 1 µM pan-caspase inhibitor Z-VAD-FMK (Sigma), detached by incubation in a trypsin/EDTA solution, washed twice with cold PBS and resuspended in 100 µl PBS, followed by incubation with 0.25 µg/ml FITC-conjugated Annexin V and 1 µg/ml PI in PBS for 15 min at room temperature in the dark. Before flow cytometry (BD LSRII FACS), cells were washed twice with 1x PBS and resuspended in 500 µl PBS. Data were analyzed using the FACS diva version 6 software.

### Western blot analysis

Immunoblotting was performed with whole cell lysates, fractionated lysates, isolated nuclei or immunoprecipitation samples using the NuPAGE system according to the manufacturer's recommendations (Invitrogen). For preparation of whole cell lysates cells were washed with PBS and lysed in Laemmli lysis buffer (67 mM Tris-HCl pH 6.8, 0.7% SDS). Subcellular fractionation was performed using the ProteoExtrac Subcellular Proteome Extraction Kit (Calbiochem) following the manufacturer's protocol. Immunoprecipitations were performed using standard protocols. The following antibodies were used: anti-penta His (Qiagen), anti-FlagM2 (Sigma), OXPHOS complex I subunit 39 k (Invitrogen), anti-S6 ribosomal protein (Cell Signalling), anti-Tfam, anti-Tom20, anti-Ubf1, anti-Fibrillarin (Santa Cruz Biotechnology). The Alexafluor 680-conjugated secondary antibodies from different species were purchased from Invitrogen. The IRD-800 antibodies were purchased from Rockland. The polyclonal antibody against NOA1 was raised in rabbits by Eurogentec (Liege, Belgium) and detects specifically precursor and mature NOA1 protein (Figure S1 in File S1).

### Immunochemistry

Cells were washed with PBS and fixed with 4% paraformaldehyde for 10 min. Cells were permeabilized with 0.3% Triton X-100 in PBS for 10 min and washed with PBS before addition of the primary antibody and incubation overnight. Cells were stained for 1 hour with secondary antibody after washing with PBS. DAPI was applied together with the last wash. NOA1-rabbit polyclonal antibody was purchased from Eurogentec. The cy3 and cy2-labeled secondary antibodies were purchased from Dianova. The Alexafluor 488- and 594-conjugated secondary antibodies were purchased from Invitrogen. Microscopy was performed with a Zeiss Z1 microscope with Apotome and a Zeiss Confocal Laser Scanning Microscope LSM710. Data were analyzed using CLSM software from Zeiss. Nuclear import assays were established based on previously published protocols [22], [23]. Details are described in supplementary materials.

### Recombinant NOA1-His6 protein

In this study, recombinant mouse NOA1-His6 protein lacking the mitochondrial targeting sequence (aa 1–17) was used, which was generated as described before [32].

### ClpXP protein purification and *in vitro* degradation assay

*E. coli* ClpX was purified as described in [24], *E. coli* ClpP-His_6_ [25] and *H. sapiens* ClpP-His_6_ were purified as described [26]. The predicted mature form of mouse ClpX (BC061153, aa 66–634) was expressed from pET28b with N-terminal fusion to His_6_SUMO, by induction with 0.5 mM IPTG at 22°C for 4 h in BL21(DE3) *E. coli*. Degradation of NOA1 (3 nM) *in vitro* was assayed in the presence of 0.3 nM ClpX hexamer, and 0.8 nM ClpP 14-mer, in 25 mM Hepes pH 7.6, 100 mM KCl, 5 mM MgCl_2_, and 10% glycerol using an ATP regenerating system (5 mM creatine phosphate and 50 mg/mL creatine kinase, 4 mM ATP). Degradation was performed at 30°C. Samples from different time points were taken by pipetting aliquots of the incubation mixture into Laemmli SDS-PAGE loading buffer. Degradation products were separated by SDS-PAGE, stained with Sypro Orange, and imaged with a Typhoon scanner (GE Healthcare).

## Results

### The NOA1 protein is transported into mitochondria and the nucleus

**Figure 1:**
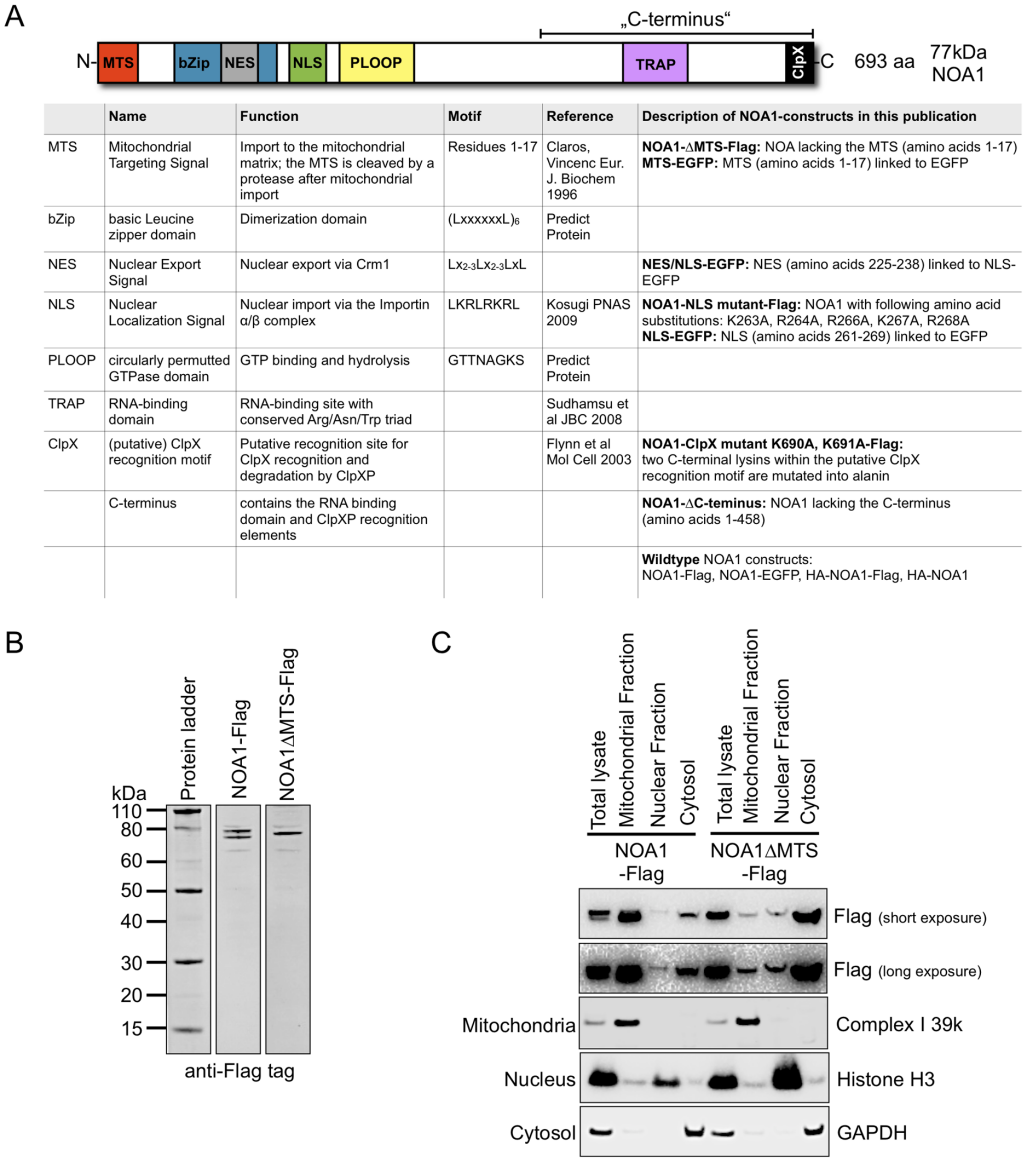
Unprocessed NOA1 precursor protein is localized in the nucleus. (A) Schematic representation of functional domains of the NOA1 protein and description of constructs used in the study. (B) Western blot analysis of C2C12 cells transfected with a NOA1 expression construct. The antibody against the C-terminal Flag-epitope detects the unprocessed precursor protein and the shorter processed protein present in mitochondria. Deletion of the N-terminal mitochondrial targeting sequence (MTS) prevents processing. (C) Subcellular fractionation of HEK293 cells expressing NOA1-Flag and NOA1ΔMTS-Flag reveals presence of unprocessed precursor proteins in the nuclear fraction marked by Histone H3 and the processed mature form in the mitochondrial fraction marked by Complex I 39 k. GAPDH served as marker for the cytosolic fraction.

The mitochondrial, multi-domain GTPase NOA1 (Fig. 1A) contains a classical N-terminal mitochondrial targeting sequence (MTS) that is processed during mitochondrial import resulting in a shorter mature protein. To analyze the functional relevance of the potential MTS we deleted the first 17 amino acids of the NOA1 precursor protein. Deletion of the MTS prevented processing of NOA1 as indicated by the presence of a single band in Western blot analysis after transfection of NOA1ΔMTS (Fig. 1B). *In silico* analysis (cNLS mapper [27]) predicted a classical bipartite nuclear localization signal in the amino acid sequence of NOA1 (Fig. 1A). To analyze whether NOA1 is transported into the nucleus, different subcellular fractions of HEK293 cells were prepared and analyzed for the presence of unprocessed and processed NOA1 by Western blotting. The processed NOA1 protein was exclusively located in the mitochondrial fraction, which is marked by the mitochondrial respiratory complex protein Complex I 39 k. In contrast, we found enrichment of the unprocessed NOA1 precursor protein in the nuclear fraction identified by the presence of Histone H3. Importantly, the nuclear fraction was free of mitochondrial contaminations as demonstrated by the absence of Complex I 39 k signals. Removal of the MTS prevented specific mitochondrial uptake and resulted in the accumulation of ΔMTS-NOA1-Flag in the cytosolic and nuclear fraction. The weak ΔMTS-NOA1-Flag signal in the mitochondrial fraction is most likely due to contamination by nuclear proteins as indicated by the presence of low amounts of Histone H3 (Fig. 1C). The data demonstrate that unprocessed NOA1 precursor protein is abundant in a protein fraction enriched for nuclear proteins while the processed form after cleavage of the mitochondrial targeting sequence exclusively resides in mitochondria.

### A fraction of endogenous NOA1 protein forms puncta in the nucleus

**Figure 2:**
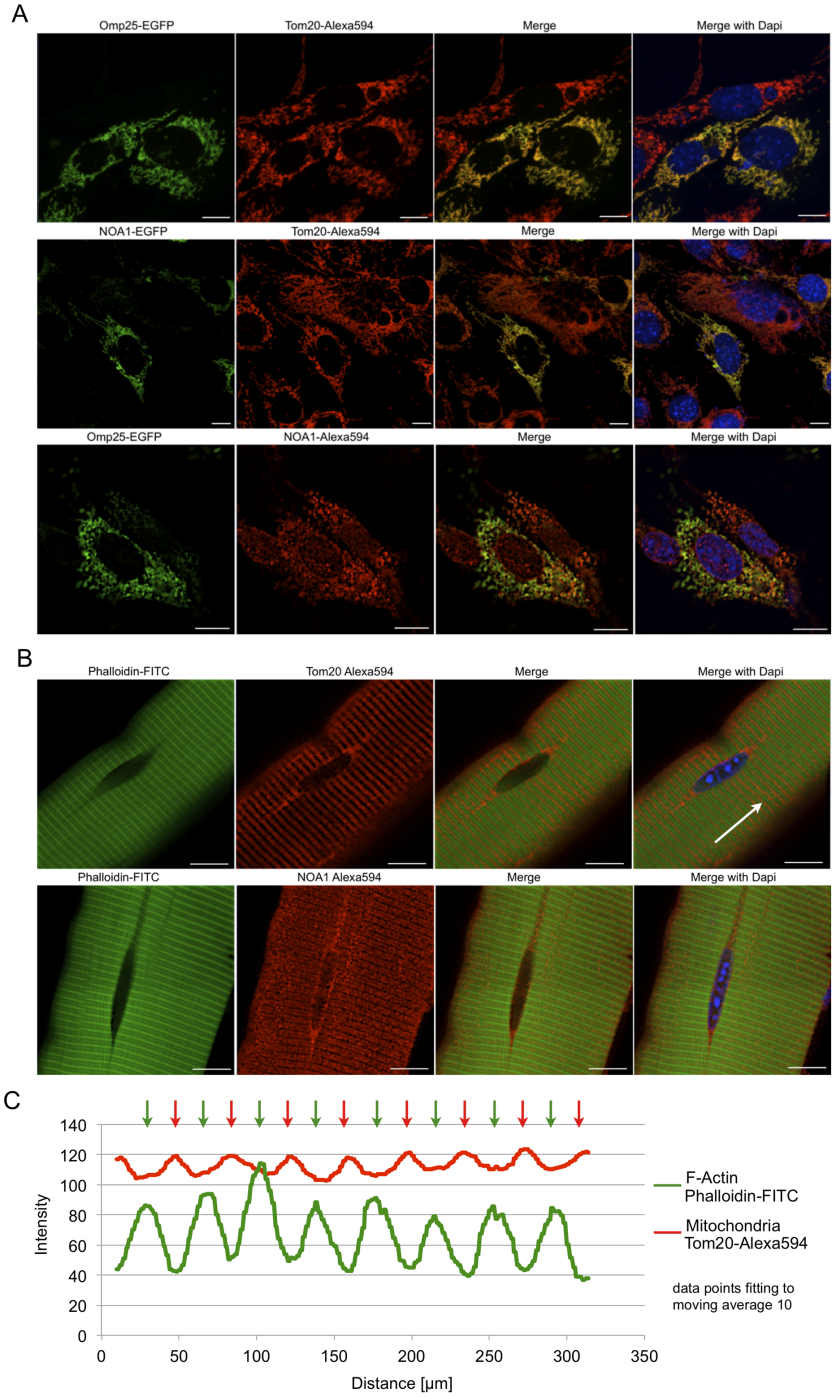
A fraction of endogenous NOA1 is found in the nucleus of C2C12 cells and primary mouse myofibers. (A) Immunofluorescence staining of NOA1-transfected C2C12 cells. Mitochondria were visualized by antibody staining with the outer mitochondrial membrane markers Tom20 and Omp-25. Overexpressed NOA1-EGFP mainly localizes in mitochondria as shown by co-localization with the mitochondrial markers Tom20 and Omp-25. Endogenous NOA1 protein detected with a rabbit polyclonal anti-NOA1 antibody also localizes with mitochondria although a fraction of NOA1 is present in the nucleus. (B) Mitochondria in primary myofibers show a striped pattern alternating with F-actin. In primary mouse myofibers endogenous NOA1 presents a striated pattern similar to Tom20 and is also seen in punctae in myonuclei. (C) Diagram of the alternating F-actin/Phalloidin-FITC and Mitochondria/Tom20-Alexa594 fluorescence intensities shown in (B). The arrow highlights the analyzed region. Data points were fitted by a line with moving average of 10. Confocal images, scale bars: 10 µm.

Next, we investigated the subcellular distribution of overexpressed and endogenous NOA1 protein in different cell lines and primary myofibers by immunofluorescence. We either used co-expression of the mitochondrial marker protein Omp25-EGFP or co-immunostaining with the Tom20 antibody, which both label the mitochondrial network of cells (Fig. 2A). NOA1-EGFP co-localized with Tom20 confirming the predominant mitochondrial localization of NOA1. Interestingly, immunostaining for endogenous NOA1 revealed that a fraction of NOA1 formed puncta that co-localized with mitochondrial Omp25-EGFP and additional foci in the nucleus confirming our results from the subcellular fractionation data (Fig. 1C). To confirm the nuclear localization of endogenous NOA1 under basal conditions we analyzed freshly isolated mouse myofibers. Immunofluorescence staining revealed the presence of endogenous NOA1 in myonuclei while Tom20 was completely absent from myonuclei (Fig. 2B). In contrast, co-localization of NOA1 and Tom20 was seen in areas enriched in mitochondria surrounding the myonuclei and throughout the myofiber (Fig. 2B). In cultured cell lines such as C2C12 mitochondria form a network-like structure (Fig. 2A) while myofibers contain a highly ordered F-actin cytoskeleton that leads to an alternating, linear arrangement of mitochondria (Fig. 2B, C).

### Newly translated NOA1 precursor accumulates in the nucleus

**Figure 3:**
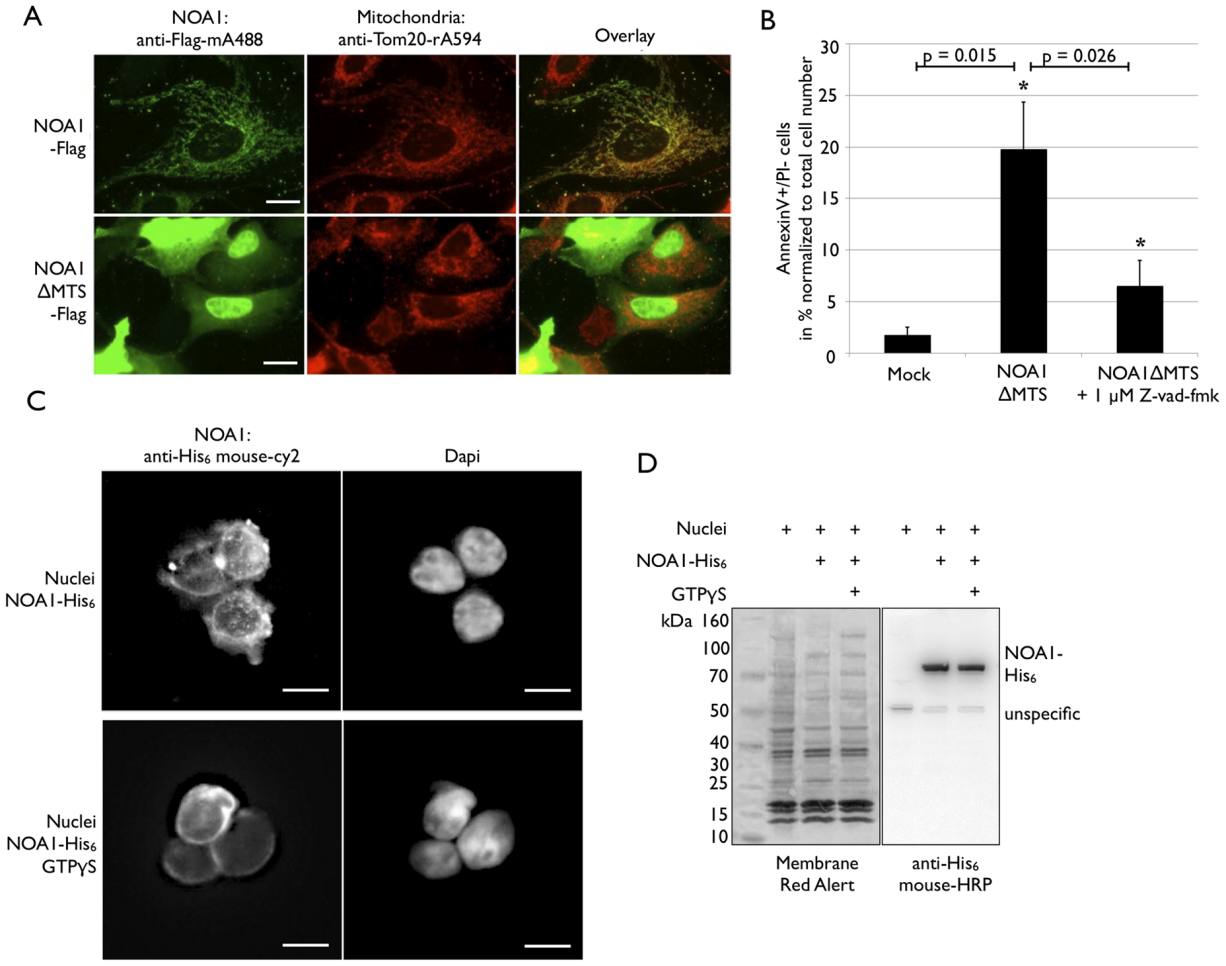
NOA1 carries a functional nuclear localization signal mediating GTP dependent active nuclear import. (A) Immunofluorescence staining of NOA1-transfected C2C12 cells. Wild type NOA1 co-localizes with the mitochondrial marker protein Tom20 while deletion of the N-terminal MTS causes accumulation of EGFP-tagged NOA1 protein in the nucleus. (B) Overexpression of NOA1ΔMTS in C2C12 leads to apoptosis, which is partially rescued by addition of 1 µM pan-caspase inhibitor Z-VAD-FMK. A p-value <0.05 was considered significant. (C) Optical section of isolated RAW264.7 nuclei stained with an antibody against recombinant NOA1-His_6_. NOA1 is imported into the nuclei in a GTP-dependent manner. Import is inhibited by addition of the non-hydrolysable GTP analogue GTPγS to the nuclear import assay. (D) Western Blot analysis of RAW264.7 nuclei after incubation with NOA1-His_6_ in nuclear import assays demonstrates equal loading of experiment and control nuclei with recombinant protein. Scale bars: 10 µm.

Cellular fractionation and immunofluorescence staining revealed that NOA1 is present in two different cellular compartments (i.e. nucleus and mitochondria) although the bulk of the protein is localized in mitochondria. To investigate whether the subcellular localization of NOA1 is subject to dynamic regulation we established an assay system based on the fusion of NOA1 to EGFP and Flag. We confirmed that transfected NOA1-EGFP and NOA1-Flag predominantly localize to the mitochondrial compartment (Figure S2A in File S1). Next, we screened a library of pharmacological active compounds (LOPAC, Sigma, LO1280) for a molecule that might change has the subcellular localization of NOA1. Each compound of the library was applied to NOA1-EGFP expressing cells at a concentration of 100 µM, followed by assessment of the subcellular distribution of EGFP fluorescence after six hours of incubation. Interestingly, four (flunarizine dihydrochloride, 5-(N,N-Dimethyl)amiloride hydrochloride, quazinone, trequinsin hydrochloride) out of 1280 screened compounds promoted accumulation of NOA1 in the nucleus (Table S1 in File S1). The most profound effect was induced by flunarizine (Figure S2B in File S1), which changes cellular Ca2+ homeostasis by inhibition of Ca2+ channels but also exerts additional dose-dependent effects. The effect of flunarizine corresponds well to the known effects of increased intracellular Ca2+, which prevents nuclear shuttling whereas Ca2+ chelators enhance nuclear translocation [28]. Additionally, flunarizine has been described before to induce nuclear translocation of Nrf2 probably by potential inhibition of nuclear export [29], [30]. To analyze whether flunarizine treatment primarily influences transport of newly translated precursor protein or causes redistribution of already existing protein from mitochondria to nuclei, we administered cycloheximide (CHX) to block translation. CHX alone had no effects on the localization of NOA1-EGFP in mitochondria (Figure S2C in File S1). However, concomitant treatment of cells with flunarizine and CHX abolished nuclear accumulation of NOA1-EGFP (Figure S2D in File S1) indicating that only newly translated NOA1 precursor protein still carrying the mitochondrial targeting sequence is routed into nuclei. Interestingly, nuclear accumulation of overexpressed NOA1-EGFP resulted in massive increase of apoptosis after prolonged flunarizine treatment. Accompanying treatment with CHX prevented cellular apoptosis indicating that nuclear accumulation of NOA1 but not additional effects of flunarizine was responsible for the increase in programmed cell death (Figure S2F in File S1). In a secondary screen, we confirmed the nuclear accumulation of endogenous NOA1 using selected LOPAC compounds (Table S1 in File S1). Interestingly, induction of cell death by NOA1 was dependent on the amount of NOA1 protein trapped in the nucleus since nuclear accumulation of endogenous NOA1, e.g. by flunarizine treatment of non-transfected cells, was not sufficient to cause apoptosis (Figure S2C in File S1). Since previous results indicated that inactivation of NOA1 protect cells from apoptosis [7], we further analyzed the relation of nuclear accumulation of NOA1 protein and apoptosis. Transfection of a Flag-tagged NOA1ΔMTS construct resulted in prominent accumulation of NOA in the nucleus completely abolishing mitochondrial import (Fig. 3A) and induced apoptosis in 20% of the total cell population as indicated by Annexin V and propidium iodide staining followed by FACS analysis. Since we achieved a ∼30% transfection efficiency we calculated that ∼66% of all NOA1ΔMTS expressing cells undergo apoptosis (Fig. 3B). Induction of apoptosis caused by nuclear accumulation of NOA1ΔMTS was efficiently repressed by addition of the pan-caspase inhibitor Z-VAD-FMK during transfection (Fig. 3B) In conclusion nuclear accumulation of high levels of NOA1 protein induces cell death. Such nuclear accumulation can be achieved by overexpression of NOA1ΔMTS or by treatment of NOA1-EGFP expressing cells with flunarizine. It will be interesting to address the underlying mechanism of these observations in further studies.

### Nuclear import of NOA1 is directed by a canonical nuclear localization signal

NOA1 has a size of 77 kDa, which is too large for passive diffusion through nuclear pores. Hence, we assumed that nuclear import is accomplished by the importin system acting in concert with the NLS in NOA1. To further investigate active nuclear import of NOA1 we established an *in vitro* nuclear import system using isolated mouse macrophage (RAW264.7) nuclei and recombinant mouse NOA1 protein. Immunofluorescence staining (Fig. 3C) and Western blot analysis (Fig. 3D) revealed that NOA1 is imported and retained in nuclei in in the presence of the nuclear export blocker leptomycin-B [31]. Addition of non-hydrolyzable GTPγS instead of GTP, which is required by the importin system, prevented nuclear import (Fig. 3C). Since GTPγS blocks import but does not prohibit interaction of the importin machinery with imported proteins we observed coating of nuclei with NOA1 protein (Fig. 3C). As a consequence, GTPγS-mediated inhibition of NOA1 import was not detectable by Western blot analysis, which included proteins bound but not necessarily processed by the importin machinery. Western blot analysis confirmed that equal amounts of NOA1 were used for the GTPγS and the control experiment (Fig. 3D).

### NOA1 localizes to nucleoli and interacts with UBF1 in an RNA and DNA independent manner

**Figure 4:**
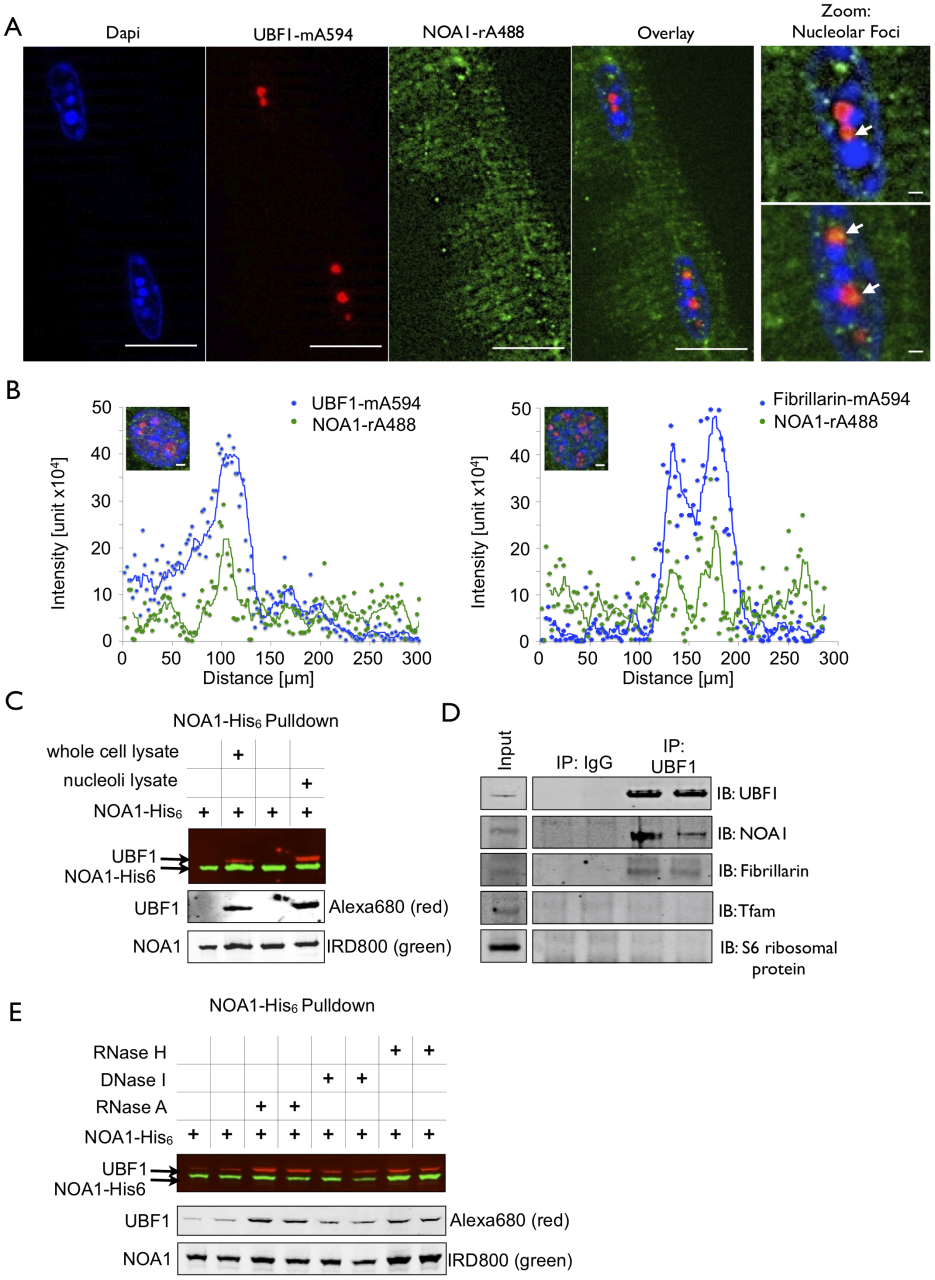
A fraction of NOA1 localizes in the nucleolus and interacts with UBF1. (A) Immunofluorescence staining of endogenous NOA1 in primary mouse myofibers reveals that a fraction of NOA1 is localized in the nucleolus. (B) Quantitative evaluation of confocal images of NIH 3T3 fibroblasts stained with antibodies against NOA1, UBF1 and Fibrillarin. NOA1 and UBF1 or NOA1 and Fibrillarin are co-localized in the nucleolus. Moving average smoothing was applied to fit data points into curves. (C) Pull-down assays demonstrating interaction of NOA1 and UBF1. Recombinant NOA1-His_6_ protein was loaded on Ni-NTA beads and mixed with whole cell or nucleoli lysates from C2C12 cells to pull down interacting proteins. (D) Co-immunoprecipitation of NOA1-His_6_ with endogenous UBF1 from C2C12 lysates. (E) Addition of RNAse H or RNAse A to pull-down assays increased the efficiency of the interaction between NOA1 and UBF1 while addition of DNAse I had no effect. Scale bars: 10 µm, Zoom scale bars: 1 µM.

To analyze whether NOA1 localizes to specific sites within nuclei we performed immunostainings of nuclei located in isolated mouse skeletal muscle myofibers. Endogenous NOA1 protein was present within distinct foci of myonuclei, which were identified as nucleoli based on UBF1 co-staining (Fig. 4A). Similar results were also obtained in other cell types such as fibroblasts (data not shown). Quantitative analysis of confocal immunofluorescence data obtained in NIH 3T3 cells revealed that the major peaks produced by NOA1-staining co-localized with peaks from UBF1 and fibrillarin, another common marker for nucleoli (Fig. 4B). Since NOA1 and UBF1 showed a striking co-localization in nucleoli we tested whether both proteins might interact directly. Pull-down assays using recombinant NOA1-His_6_ protein loaded on Ni-NTA beads and whole cell or nucleoli lysate revealed a physical interaction of NOA1 and UBF1 (Fig. 4C). To confirm this finding, we performed co-immunoprecipitation experiments using antibodies directed against endogenous UBF1. We found that UBF1 is present in a complex containing NOA1 and fibrillarin. No interaction was scored for Tfam and S6 ribosomal protein, which were used as negative controls (Fig. 4D). Since NOA1 contains a RNA binding domain, we next asked whether the interaction between NOA1 and UBF1 requires RNA or DNA. Addition of RNAses and DNAse to the pull-down assays did not compromise the interaction between NOA1 and UBF1 but resulted in a mild increase of UBF1 in pull-down assays, which might indicate that RNA restricts interaction of NOA1 with UBF1 (Fig 4E). We recently reported that NOA1 binds to G-quadruplex RNA [32]. Hence, it is tempting to speculate that increased concentrations of G-quadruplex RNA ligands limit binding of NOA1 to UBF1.

### NOA1 contains a leptomycin-B sensitive nuclear export signal that mediates nucleo-cytoplasmic shuttling

**Figure 5:**
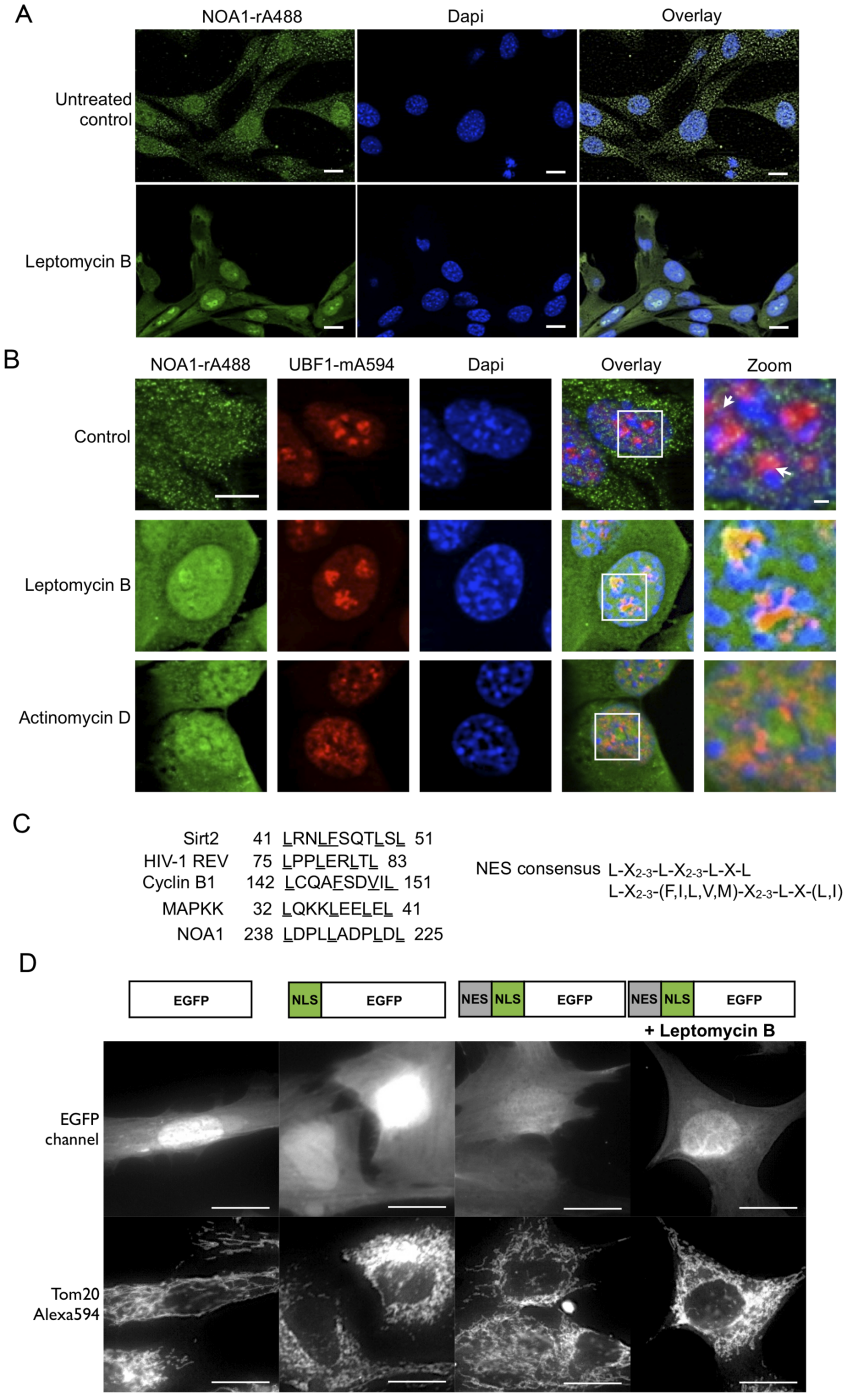
Nuclear export of NOA1 is mediated by a Crm1-dependent Nuclear Export Signal (NES). A) Immunofluorescence staining of endogenous NOA1 in C2C12 cells. One-hour treatment with 10 ng/ml leptomycin-B leads to accumulation of NOA1 in nuclear foci. (B) Nuclear NOA1 is localizes in distinct foci of UBF1-stained nucleoli and accumulates after leptomycin-B (10 ng/ml) and actinomycin D (1 µg/ml) treatment. (C) Amino acid sequences of the nuclear export sequence (NES) of different proteins including NOA1. (D) Different cellular targeting sequences of NOA1 direct cellular localization of EGFP fusion proteins. Leptomycin-B treatment (10 ng/ml) leads to increased accumulation of NLS-NES-EGFP fusion protein in nucleus. Tom20 staining was used to identify mitochondria. Scale bars: 10 µm, zoom scale bar: 1 µM.

So far, we demonstrated that the nuclear import of NOA1 is directed by a canonical nuclear localization signal resulting in accumulation of NOA1 in nucleoli. To learn more about the mechanism that are responsible for the export of NOA1 from nuclei and subsequent import into mitochondria, we asked whether nuclear export of NOA1 depends on Crm1, which is a key component of the nuclear export machinery. Since Crm1 is covalently modified and inactivated by leptomycin-B [31] we treated C2C12 myoblasts with leptomycin-B. Immunofluorescence staining of endogenous NOA1 protein revealed a punctate pattern (Fig. 2) indicating a strong accumulation of endogenous NOA1 in nucleoli of C2C2 cells upon nuclear export blockade (Fig. 5A). Inhibition of nuclear export resulted in a homogenous distribution of NOA1 signal throughout the cell, which contrasted to the discrete punctate pattern in the control situation (Fig. 5A). Similar results were obtained in fibroblasts. Again, the NOA1 protein appeared to be homogeneously distributed throughout the nucleoplasm and the cytoplasm when nuclear export was blocked by leptomycin-B treatment (Fig. 5B). Inhibition of transcription by actinomycin D phenocopied leptomycin-B induced accumulation of NOA1 in the nucleus and nucleoli suggesting that nuclear export and subsequent import of NOA1 into mitochondria requires active transcription (Fig. 5B).

The observation that nuclear export of NOA1 was dependent on Crm1 prompted us to search for a corresponding putative nuclear export signal (NES). In fact, we detected a NES motif positioned in reverse order within the predicted leucine zipper domain (Fig 5C). EGFP lacking a specific targeting signal is found in the cytosol and also causes bright fluorescence in the nucleus. Fusion of the NOA1-NLS to EGFP did not cause dramatic changes in the distribution of EGFP while addition of the putative NOA1-NES to NLS-EGFP clearly reduced nuclear fluorescence. Most likely, the increased nuclear export of NLS-NES-EGFP compared to NLS-EGFP was responsible for the reduced accumulation in nuclei (Fig. 5D). Accordingly, treatment of cells transfected with the NLS-NES-EGFP construct with leptomycin-B increased EGFP fluorescence in the nucleus confirming functionality of the putative NOA1-NES (Fig. 5D).

### The RNA binding domain containing C-terminus and prior nucleolar localization are critical for mitochondrial import of NOA1

**Figure 6:**
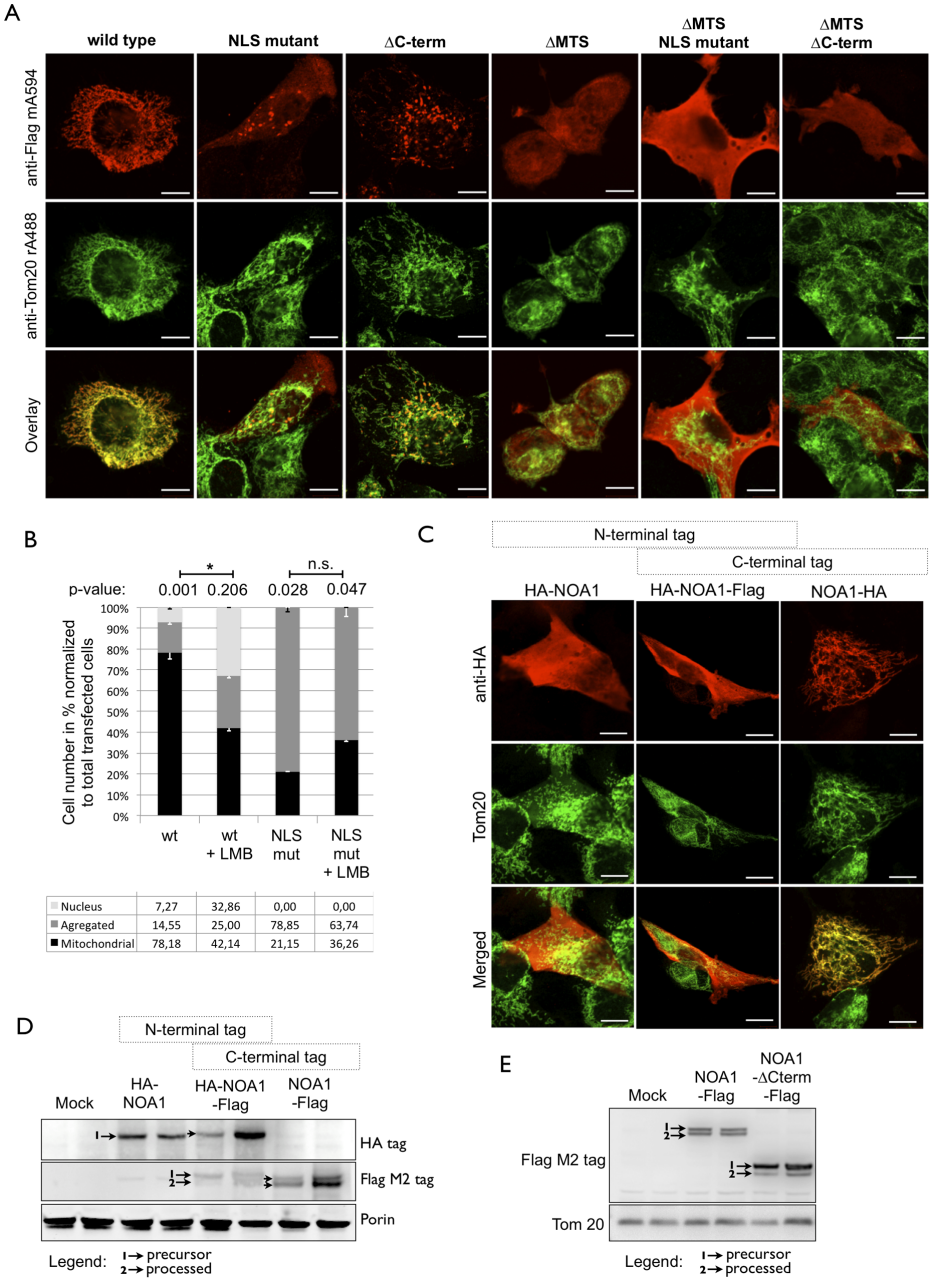
Nuclear localization and the 25 kDa C-terminus is essential for subsequent mitochondrial import of NOA1. (A) Immunofluorescence staining of different Flag tagged NOA1 proteins transfected into C2C12 cells reveals requirement of the NLS and the C-terminus of NOA1 for mitochondrial import. Deletion of the MTS abolishes mitochondrial localization leading to accumulation of NOA1 in the nucleus. Mutation of the NLS in the ΔMTS mutant confirms that the NLS is necessary for nuclear localization. Mitochondria were identified by co-staining for Tom20. (B) Quantitative evaluation of the subcellular dynamics of wild type and mutant NOA1 protein. Wild type NOA1 protein shows a predominant “Mitochondrial” localization. ΔMTS-NOA1 shows a predominant “nucleus” accumulation. NOA1-NLS mutants show a mixed localization as indicated by the term “aggregated”. Wild type NOA1 is dynamically distributed between nucleus and mitochondria. Leptomycin-B treatment prevents nuclear export and subsequent mitochondrial import of wild type NOA1. Mutation of the NLS prevents nuclear accumulation of NOA1 after leptomycin-B treatment. (C) N-terminal tagging prevents import of NOA1 into mitochondria of NIH 3T3 cells by masking the MTS. (D) A Western blot analysis of N-terminally (HA tag) and C-terminally (Flag tag) modified versions of NOA2 is shown. (E) Deletion of the RNA-binding domain containing C-terminus prevents mitochondrial import in NIH 3T3 cells. Western blot analysis demonstrated strongly reduced processing of the MTS after truncation of the C-terminus. Scale bars: 10 µm.

To test whether localization in nucleoli is a prerequisite for mitochondrial import of NOA1, we mutated the NLS of NOA1 and assessed its subcellular localization and dynamics (Fig 6A). As expected, wild type NOA1 localized to mitochondria visualized by Tom20 staining. Mutation of the NLS in full length NOA1 strongly impaired mitochondrial localization and changed the morphology of mitochondria, which accumulated in a peri-nuclear position. NOA1 mutants carrying a mutated NLS and lacking the MTS were found outside the nucleus in the cytosol while lack of the MTS resulted in a primarily nuclear localization (Fig. 6A).

Next, we quantitatively analyzed localization of WT and NLS mutant NOA1 proteins. Cells were investigated 24 hours after transfection to avoid artifacts caused by accumulation of NOA1 in nuclei (Fig 6B). We distinguished three different localizations of NOA1: (i) mitochondrial localization; (ii) nuclear localization and (iii) “aggregated” referring to unclear or mixed subcellular localization of NOA1 probably caused by compromised mitochondrial import. About ∼80% of wild type NOA1 localized to mitochondria and only a minor fraction of 7% were found in the nucleus, which is in line with the immunostaining for endogenous NOA1 protein (Fig 4A, 5A). Leptomycin-B treatment of cells transfected with wild type NOA1 increased concentration in nuclei to ∼33% leaving ∼42% of wild type NOA1 in mitochondria (Fig 6B). Leptomycin-B also increased the aggregated localization from ∼15% to 25% probably based on the cellular stress resulting from nuclear accumulation of NOA1, which eventually causes apoptosis (Fig. 3B). As expected the NLS mutant of NOA1 did not respond to leptomycin-B because it is not imported into the nucleus. Strikingly, mutation of the NLS severely reduced mitochondrial localization of NOA1. Only 36% NLS mutant NOA1 localized to the mitochondrial compartment compared to ∼78% wild type NOA1 strongly suggesting that NOA1 needs to acquire a nuclear localization before mitochondrial import (Fig. 6B). Addition of a Flag or HA epitope to the C-terminal end of NOA1 did not interfere with mitochondrial targeting (Fig 6C) and N-terminal processing of the precursor NOA1 protein (Fig 6D). In contrast, addition of a HA-tag to the N-terminus prevented mitochondrial import, which is common for mitochondrial proteins carrying a classical N-terminal targeting sequence (Fig. 6C, Fig. 6D). Interestingly, deletion of the C-terminal part of NOA1, which contains the RNA-binding domain, disrupted correct localization to mitochondria similar to the NOA1-NLS mutant (Fig 6A). Western blot analysis confirmed that the MTS of the NOA1-ΔC-terminus mutant was not fully processed validating the failure of mitochondrial import when the RNA-binding domain was absent (Fig. 6E).

### Degradation of NOA1 by the mitochondrial matrix protease ClpXP depends on a C-terminal ClpX recognition motif

**Figure 7:**
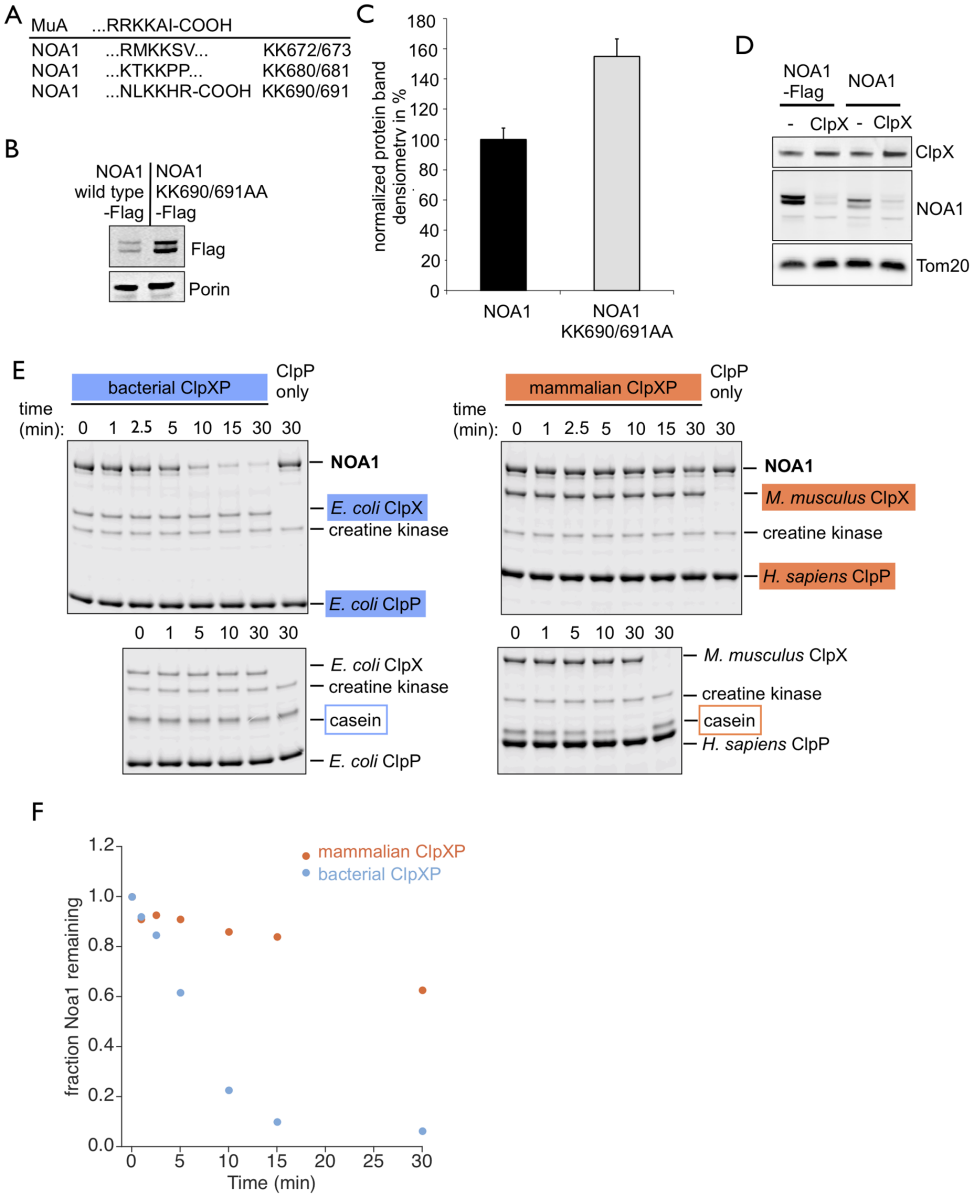
NOA1 is a substrate of the mitochondrial matrix protease complex ClpXP. (A) The C-terminus of NOA1 contains three motifs resembling known ClpX recognition motifs found in proteins of *E.coli*. (B) Exemplary Western blot analysis revealing increased concentrations of NOA1 in C2C12 cells after mutation of lysin690 and lysin691 to alanines in the proximal ClpX recognition motif. (C) Quantitative analysis of Western blots demonstrating ca. 60% stabilization of the NOA1-KK690,691AA mutant compared to wild type NOA1. Densitometries from three independent experiments were normalized to Porin. (D) Overexpression of ClpX is sufficient to promote degradation of NOA1 independent of a C-terminal Flag tag in C2C12 cell lysates. (E) Recombinant bacterial ClpXP (*E.coli* ClpXP) (top left) and mammalian ClpXP (mouse ClpX, human ClpP) (top right) cleaves recombinant NOA1 (2.5 µM) *in vitro* in a time dependent manner. Mammalian ClpXP but not by *E. coli* ClpXP cleaves α-Casein (5 µM) in vitro. (F) Quantitation analysis of Western blots shown in (E) demonstrating the degradation of NOA1 by bacterial and mammalian ClpXP *in vitro*.

Our data demonstrated dynamic regulation of the subcellular localization of NOA1 indicating the need for a tight control of NOA1 in different cellular compartments. To gain further insights into the pathways controlling NOA1 levels in mitochondria, the main site of NOA1 localization under physiological conditions, we studied its potential mode of degradation. Although some exceptions seem to exist, protein turnover within the mitochondrial matrix relies mostly on three major matrix proteases: Lon, m-AAA and ClpXP [33], [34]. Interestingly, the C-terminus of NOA1 contains a conserved motif that resembles the ClpX specific recognition motif C-motif2 found in *E. coli* substrates [35] (Fig 7A, B). To test whether turnover of NOA1 in mitochondria depends on ClpXP we mutated the two lysines in the most proximal C-motif2 of NOA1 into alanines. Insertion of the K690A and K691A mutation led to a ∼60% increase in NOA1 concentrations compared to wild type indicating stabilization of the protein (Fig 7C). Although NOA1-KK690,691AA-Flag was not entirely protected from degradation, the increased stability indicated that the C-terminal region is recognized by ClpX. Furthermore, expression of the catalytic subunit ClpX alone in NOA1 overexpressing cells resulted in a rapid depletion of NOA1 confirming that detection of NOA1 by the ClpX subunit is the limiting step for proteolytic degradation (Fig. 7D). Since the C-motif2 is present in the mammalian NOA1 protein and in prokaryotic substrates of ClpXP we tested whether recombinant *E.coli* ClpX and ClpP are able to degrade mammalian NOA1. As expected, *E.coli* ClpP alone was unable to reduce the concentration of NOA1 (Fig. 7E). In contrast, addition of the *E.coli* ClpXP complex resulted in a rapid decline of NOA1 levels within 30 min, clearly establishing NOA1 as a mammalian substrate of the conserved ClpXP system (Fig 7E). In agreement with previous reports we found that α-Casein was degraded by mammalian ClpXP, but not by *E. coli* ClpXP (Fig. 7E) (Kang et al, 2002). To reconstitute mammalian ClpXP activity *in vitro,* we used a chimeric complex consisting of mouse ClpX and human ClpP. This chimeric mammalian ClpXP complex was less efficient to degrade mouse NOA1 compared to *E.coli* ClpXP (Fig. 7E, F), which might indicate species specificities and/or indicate the existence of additional proteins assisting mammalian ClpXP.

## Discussion

NOA1 is an essential mitochondrial matrix protein, which regulates mitochondrial ribosome biogenesis and OXPHOS activity dependent on its GTPase activity [1], [2], [4]. Although several studies have addressed the function of NOA1, information about the dynamics of its subcellular localization was lacking. The current report demonstrates that NOA1 contains a functional NLS and a Crm1 dependent NES, which are responsible for active nuclear import and export. The NES is localized in a predicted leucine zipper region required for dimerization or other protein-protein interactions [1], which might be important for masking the NES thereby restricting nuclear export. A similar scenario has been described for the pivotal stress response protein NRF2 [36]. Alternatively, it seems possible that a putative NOA1 ligand or interaction partner, such the nucleolar protein UBF1, regulates accessibility of the NES and the nuclear export rate.

NOA1 shows an unusual routing within cells. We are not aware of a mitochondrial matrix protein that takes a mandatory ”detour” through another cellular compartment before embarking at its final destination although other proteins are known, which reside within the nucleus and mitochondria. However, such proteins including LRPPRC [13], [14], ELAC2 [19], CRIF1 (CR-6 interacting protein) [37], TERT [20] and p53 [21] do not necessarily enter the nucleus before ending up in mitochondria making NOA1 the founding member of a novel category of “dual targeted mitochondrial proteins”. Since the nuclear form of NOA1 still contains the mitochondrial targeting sequence, we can exclude a retrograde mito-nuclear transport. We would also like to stress that newly translated NOA1 precursor protein accumulates exclusively in the nucleus when nuclear export is blocked, which clearly argues for a sequential transport first into the nucleus and then into mitochondria ruling out the alternative usage of mitochondrial and nuclear targeting sequences.

LRPPRC, CRIF1 and NOA1 are predominantly located within mitochondria while TERT and p53 leave the nucleus mainly during cellular stress. Interestingly, the nuclear form of the GADD45 family protein CRIF1 is also involved in the regulation of cellular stress responses by negative regulation of NRF2 [38]. In contrast, its mitochondrial form regulates OXPHOS similar to NOA1 [1], [37]. However, some studies aiming at the nuclear function of CRIF1 were performed using N-terminally tagged versions of the proteins, which interferes with mitochondrial targeting increasing nuclear localization [39]. Intriguingly, nuclear LRPPRC has been reported to be part of a nuclear ribonucleoprotein complex that contains RNA. NOA1 carries an RNA binding site that interacts with G-quadruplex RNA [32]. Although very little is known about the endogenous NOA1 RNA ligand, it is tempting to speculate that NOA1 binds G-quadruplex RNA, which is highly abundant in the nucleolus. RNAse treatment resulted in a mild increase of UBF1-NOA1 interactions, which might indicate that RNA binding facilitates release of NOA1 from the nucleolar protein complex. Another hint for the relevance of binding of NOA1 to RNA in the nucleolus was the effect of actinomycin D, which retained NOA1 in the nucleus similar to the nuclear export blocker leptomycin-B. This observation supports the idea that release of NOA1 from the nucleolus might depend on newly transcribed RNA ligands, although we presently lack firm evidence for such a mechanism. Under any circumstances, the alternative localization of NOA1 might indicate different functions in the nucleus versus mitochondria, affect bioavailability in each compartment or allow communication between both organelles.

**Figure 8:**
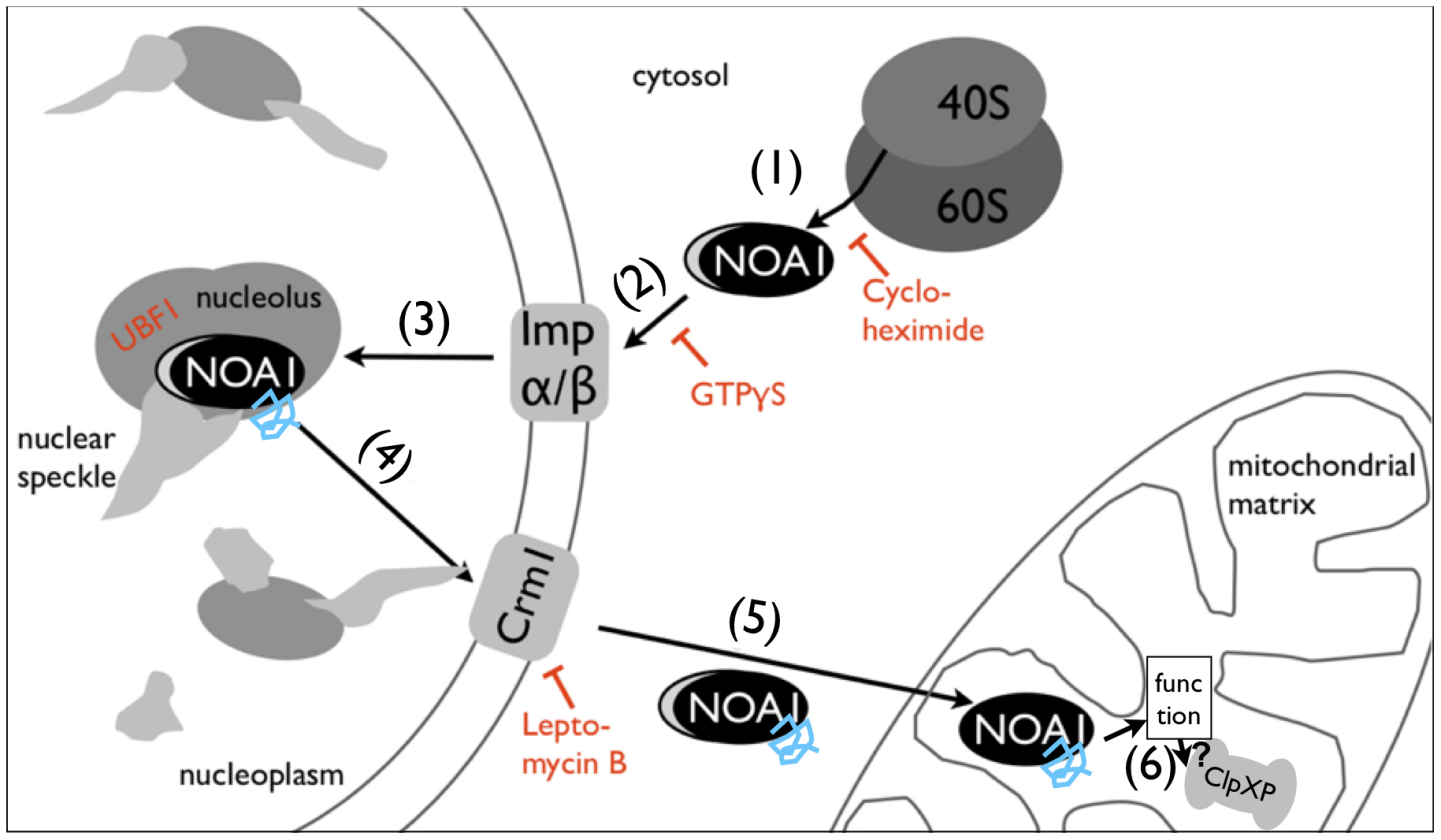
Model of the intracellular routing of NOA1. NOA1 is a nuclear encoded protein translated in the cytosol. The unprocessed precursor NOA1 protein (1) is imported into the nucleus in a NLS dependent manner mediated by the importin system, which requires GTP. (2). NOA1 localizes to the nucleolus and interacts with the UBF1 protein (3). NOA1 binds G-quadruplex RNA, which destabilizes interaction of NOA1 with the UBF1 protein complex followed by NES dependent Crm1 mediated nuclear export (4). Following nuclear export NOA1 is imported into the mitochondrial matrix where the mitochondrial targeting sequence is removed (5). The matrix protease complex ClpXP most likely mediates degradation of NOA1 in mitochondria (6) although the mammalian ClpXP complex was less efficient to degrade NOA1 compared to bacterial ClpXP.

The finding that the mitochondrial matrix protease complex ClpXP mediates degradation of NOA1 further supports our model of a unidirectional nucleo-mitochondrial pathway (Fig. 8). Once NOA1 reaches its final destination, the mitochondrial matrix, it will be degraded on-site by ClpXP after fulfilling its role as a regulator of mitochondrial protein synthesis and OXPHOS. We reason that similar nucleo-mitochondrial transport pathways might also be employed by LRPPRC, CRIF1 and other proteins, which have not been studied in this respect. In case of NOA1, disruption of the nucleo-mitochondrial transport pathway is detrimental for the cell. Accumulation of NOA1 in the nucleus presents a strong trigger for apoptosis, which can be blocked by pan-caspase inhibitors. Further studies will show whether this novel means of induction of apoptosis is used by the cell under pathophysiological conditions, e.g. by actively blocking export of NOA1 from the nucleus.

## Supporting Information

File S1 **Supporting table and figures. Table S1,** Screening of nuclear compounds initiating nuclear accumulation of NOA1. A panel of 1280 compounds (LOPAC1280 panel, Sigma Aldrich) was screened for factors causing accumulation of NOA1 in nuclei of C2C12 cells. The primary screen for compounds was evaluated by analysis of nuclear fluorescence caused by transfected NOA1-EGFP six hours after treatment with 100 µM concentrated substance in DMSO. 3.2% (41/1280) of the compounds induced increased nuclear accumulation of NOA1. 30.08% (385/1280) of the compounds induced cell death at a concentration of 100 µM. Substances causing severe toxic effects on C2C12 cells were excluded. Effects of compounds were compared to negative controls (1% DMSO and water), which did have no effects on the subcellular localization of NOA1-EGFP. All 41 compounds that were testing positive were subjected to a secondary screen based on the accumulation of endogenous NOA1 in nuclei of C2C12 cells. 17 out of 41 (1.33% of total) retested compounds consistently initiated accumulation of endogenous NOA1 in the nucleus of more than 10% of the cells. Four substances (trequinsine, quazinone, flunarizine and amiloride) were further analyzed. Flunarizine yielded the strongest effects and was therefore employed for subsequent studies. **Figure S1,** Rabbit polyclonal anti-NOA1 antibody is specific for precursor and mature NOA1 protein. To assess specificity of the rabbit polyclonal anti-NOA1 antibody used in this study we expressed C-terminally Flag tagged NOA1 in C2C12 myoblasts and analyzed the protein lysates by Western blotting. Flag antibody detected the larger precursor and the shorter, mature, N-terminally processed NOA1 protein. Similar result was obtained with the antibody raised against NOA1. Although highly specific, the endogenous NOA1 protein expression was below threshold for detection by Western blot. **Figure S2,** Flunarizine induces accumulation of NOA1 in the nucleus. (A) Immunofluorescence staining of NOA1-transfected C2C12 cells. NOA1 mainly localizes in mitochondria as demonstrated by co-localization with the mitochondrial marker protein Tom20. (B) Six hour treatment with flunarizine (100 µM), identified by screening a LOPAC library for compounds altering subcellular localization of NOA1, leads to rapid accumulation of NOA1-EGFP in the nucleus of C2C12 cells. (C) FACS analysis of non-transfected C2C12 cells after Flunarizine treatment (100 µM for 6 hours) shows no increase of apoptosis. (D) Inhibition of translation by 10 ng/ml Cycloheximide (CHX) does not affect mitochondrial localization of NOA1-EGFP. (E) Combined treatment with Flunarizine (100 µM) and CHX (10 ng/ml) prevents accumulation of NOA1 in the nucleus indicating that only newly translated NOA1 is targeted to the nucleus. (F) Combined CHX and flunarizine treatment of NOA1-EGFP transfected C2C12 cells reduces the percentage of NOA1-EGFP positive non-viable cells compared to flunarizine treatment alone. Cell viability was assessed by counting the number of attached and non-rounded EGFP-positive cells. Scale bars: 10 µm.
(PDF)
