# Supplementary material for: NOA1, a Novel ClpXP Substrate, Takes an Unexpected Nuclear Detour Prior to Mitochondrial Import
Source: PLoS One. 2014 Jul 29;9(7):e103141. doi: 10.1371/journal.pone.0103141 (PMC4114565; doi:10.1371/journal.pone.0103141)

Supporting Information File S1

NOA1, a novel ClpXP substrate, takes an unexpected nuclear detour prior to mitochondrial targeting

Natalie Al-Furoukh[1], Julia R. Kardon[2], Marcus Krüger[1], Marten Szibor[1], Tania A. Baker[2] and Thomas Braun[1#]

[1]Department of Cardiac Development and Remodeling, Max-Planck-Institute for Heart and Lung Research, Bad Nauheim, Germany [2]Department of Biology, Massachusetts Institute of Technology, Cambridge, Boston, USA

[#]Corresponding author: thomas.braun@mpi-bn.mpg.de
Thomas Braun, Department of Cardiac Development and Remodeling, Max-Planck-Institute for Heart and Lung Research, Bad Nauheim, Germany, Phone: +49 6023 705-1102

Supporting Methods

Molecular Cloning

The NOA1 coding sequence (NM_019836) was amplified by PCR using proofreading high-fidelity Phusion Polymerase (Finnzymes). Two enzymatic restriction sites (5'-GGA TCC BamH1, 3'-CTC GAG SlaI) and a Flag tag (5'-GAT TAT AAG GAT GAT GAT GAT AAG-3') were introduced by primer design.

Truncation variants of NOA1 and the NOA1 NLS-mutant were produced using combinations of the following amplification primers: NOA1 forward: 5'-GGA TCC ATG CTG CCC GCG CGC CTG GCT TGC GGG CTG CTC TGC GGG; NOA1ΔMTS forward: 5'-GGA TCC ATG CCC GCG CCG GCC GCT GCG TGC TAC GGC CCA GCA CGG-3'; NOA1-Flag stop reverse: 5'- TAA CTC GAG TCA CTT ATC ATC ATC ATC CTT ATA ATC TCT GTG CTT CTT CAG GTT G-3'; NOA1-ΔC-terminus-Flag stop reverse: 5'- TAA CTC GAG TCA CTT ATC ATC ATC ATC CTT ATA ATC GTC GGC ATC AAA CTC AAA G-3'; NOA1-NLS mutant (K263A, R264A, R266A, K267A, R268A) forward: 5'-ACG CTC CCG GCT ACT TGG CGG CGC TGG CGG CGG CGC TGT GGG ACG ATT GCA T-3'; NOA1-NLS mutant (K263A, R264A, R266A, K267A, R268A) reverse: 5'-ATG CAA TCG TCC CAC AGC GCC GCC GCC AGC GCC GCC AAG TAG CCG GGA GCG T-3'; NOA1-ClpX mutant (K690A, K691A) reverse: 5'-CTC GAG TCA TCT GTG GGC GGC CAG GTT GTG CAC-3'. Codons leading to amino acid exchanges are underlined.

Using Phusion polymerase (Finnzymes) a primer specific PCR product was generated following the manufacturer's protocol. In short, 1 µl of template DNA was mixed with 12.8 µl H_2O, 4.0 µl 5x HF buffer, 0.4 µl dNTPs (10 mM), 0.5 µl forward oligonucleotide (10 pmol/µl), 0.5 µl reverse oligonucleotide (10 pmol/µl), 0.6 µl DMSO and 0.2 µl Phusion Polymerase. Amplification was performed in an Eppendorf light cycler using the following program: 1-94°C, 30 sec, 2-94°C, 10 sec, 3-68°C, 15 sec, 4-72°C, 25 sec, 5-72°C 5 min; 2-4 repeat 26 times.

In case of the NOA1 NLS-mutant, two fragments were produced by using the following primer combinations: NOA1 sense/NOA1 NLS mutant antisense and NOA1 NLS mutant sense/NOA1 Flag stop reverse. The two fragments were subjected to an overlap PCR with the Phusion polymerase. The Phusion PCR product was extracted from an 1% agarose gel by using the PCR Clean up Kit (Macherey-Nagel). The eluate was subjected to A-overhang synthesis by incubating 43 µl of eluate with 5 µl 10x PCR buffer (5PRIME), 1 µl dATP (1 mM) and 1 µl Taq Polymerase (5PRIME). The mixture was incubated for 20 minutes at 70°C followed by ligation of the A-tailed PCR product into the pGemTeasy vector (Promega) using T4 DNA Ligase (Promega). Ligation was incubated overnight at 16°C and then transformed into *E.coli* Xl1Blue. The correct inserts were isolated by enzymatic digestion using the BamH1 and SlaI enzymes and subcloned to the target expression vector pcDNA5/TO (Invitrogen) or to the pcDNA5/TO vector containing a HA-tag (5'-TAC CCA TAC GAT GTT CCA GAT TAC GCT-3') in frame, upstream of the BamH1 restriction site to generate N-terminal tagged NOA1 constructs.

The ClpX coding sequence (NM_011802) was amplified from cDNA derived from C57/Bl6 mouse heart. RNA was extracted with the Trizol Reagent (Life

Technologies) and reverse transcribed using the Superscript II Kit (Invitrogen). Amplification was done using the same protocol as for NOA1 with the following primer oligonucleotides: ClpX forward 5'-GGA TCC ATG TCC AGT TGC GGC GCT TGT-3', ClpX stop reverse 5'-CTC GAG TTA GCT GTT TGC AGC ATC CGC TTG AC-3'.

Linker Synthesis

To link the subcellular targeting sequences derived from the NOA1 sequence to EGFP the following oligonucleotide pairs were designed: MTS sense: 5'-GAT CCA TGC TGC CCG CGC GCC TGG CTT GCG GGC TGC TCT GCG GGC TCC GGC GGG GCC; MTS antisense: 5'-TCG AGG CCC CGC CGG AGC CCG CAG AGC AGC CCG CAA GCC AGG CGC GCG GGC AGC ATG-3'; NLS sense: 5'- GAT CCA TGT TGA AGC GGC TGC GGA AGC GGC TGC-3'; NLS antisense: 5'- TCG AGC AGC CGC TTC CGC AGC CGC TTC AAC ATG-3'; NLS-NES sense: 5'-GAT CCA TGT TGA AGC GGC TGC GGA AGC GGC TGC TAG ACC TGC CGG ACG CCC TGC TAC CCG ACC TGC CCA AGC TGC-3'; NLS-NES antisense: 5'- TCG AGC AGC TTG GGC AGG TCG GGT AGC AGG GCG TCC GGC AGG TCT AGC AGC CGC TTC CGC AGC CGC TTC AAC ATG-3'. Each sense/antisense pair was subjected to a phosphorylation step and an annealing reaction using the following protocol. 2 µl sense oligonucleotide (100 µM), 2 µl antisense oligonucleotide (100 µM), 2 µl 10 X T4 Kinase Buffer (New England Biolabs), 1 µl 1 mM rATP, 1 µl 10 u/µl T4 Polynucleotide Kinase (New England Biolabs) and 12 µl H_2O were incubated for one hour at 37°C followed by the annealing reaction: 3 min at 95° C and then a slow cool down with 0.8 °C steps per cycle during 99 cycles. The annealed oligonucleotides have a start codon (5'-ATG-3') and overhangs fitting to BamH1 (5') and Sla1 (3') enzymatic restriction sites and were directly ligated into the pcDNA5/TO vector encoding EGFP downstream of the Sla1 restriction site.

Isolation of nuclei and immunoprecipitation

Nuclei were isolated by hypotonic cell lysis. Cells were incubated with 1 ml 0.1x Homogenization buffer (4 mM Tris-HCl, 2.5 mM NaCl, 0.5 mM $MgCl_2$ 0.1 mM PMSF) on ice and homogenized with a glass pestle. Then, 125 ml 2M Sucrose/TE (20 mM HEPES-KOH pH 7.5, 10 mM KCl, 1 mM EGTA, 1mM EDTA, 0.25 M Sucrose, 0.1% Fatty acid free BSA, 0.1 mM PMSF) solution was added. Nuclei were pelleted at 1200 g for 3 min at 4°C. Nuclei were further purified by repeating the procedure and washed with buffer (10 mM Tris-HCl pH 7.8, 250 mM EDTA, 2 M Sucrose) containing 0.25% NP-40. For immunoprecipitation, cells were twice washed with PBS and lysed with modified RIPA buffer (0.05% SDS, 150 mM NaCl, 1 % NP-40, 0.5 % Na-Deoxycholate, 1 mM EDTA, 5 mM Tris-HCl pH 8.0) for 30 min at 4°C. The cleared lysate was incubated with 1-5 µg antibody and 30µl FastFlow Protein G Sepharose (GE Healthcare) slurry for 1 hour. Beads were washed three times with RIPA buffer and the immunocomplex was recovered with Laemmli sample buffer (67 mM Tris-HCl pH 6.8, 0.7% SDS, 10% glycerol, bromphenol blue, 100 mM DTT) by boiling for 10 min at 95°C. All buffers contained

protease inhibitors: 1 mM PMSF, 2 mM Peptstatin A, 0.6 mM Leupeptin, 2 mM Benzamidine.

Isolation of nuclei and nuclear import assays

Isolation of nuclei was done as decribed (Igwe et al., 2009). In short, nuclei isolated from RAW264.7 mouse macrophages were used in combination with recombinant mouse NOA1-His_6 protein (Al-Furoukh et al., 2013). Cells were pelleted from a confluent 10 cm dish and incubated in 200 µl Hypotonic Nuclei Lysis Buffer (10 mM HEPES-KOH pH7.9, 0.1 mM EDTA, 2 mM MgCl2, 10 mM KCl, 1 mM DTT, 5 mM PMSF) for 10 min on ice. Cell disruption was supported by 2-4 gentle strokes in a dounce homogenizer. Cell lysis and nuclear integrity were checked microscopically by Trypan blue staining. The nuclei were pelleted at 500 g for 30 min at 4°C. The nuclear pellet was resuspended in 150 µl Nuclei Storage Buffer (50 mM HEPES-KOH pH 8.3, 0.1 mM EDTA, 5 mM MgCl2, 40 % Glycerol). For the nuclear import assay, 50µl transport buffer was mixed with 50 µl nuclei solution, 4 mM ATP, 3.25 µg Creatine phosphate, 4U Creatine phosphate kinase, ~15 µg recombinant NOA1-His_6, 44 ng/µl Leptomycin B, 0.4 mM GTP or GTPγS (Melchior, 1998). The import reaction was mixed by gentle pipetting and incubated for 30 min at RT. Nuclei were pelleted at 500 g for 10 min and washed quickly with PBS. After repelleting, nuclei were incubated in 4% paraformaldehyde solution for 10 min and washed again with PBS. The antibody staining occurred in solution with 100 µl antibody diluted 1:100 in PBS solution. Stained nuclei were mounted in Mowiol before microscope analysis.

Isolation of Nucleoli

For isolation of nucleoli from C2C12 ten 15 cm dishes were harvested by trypsinization. One hour before isolation of nucleoli fresh medium was added. Cells were washed three times with ice-cold PBS and pelleted at 218 g at 4°C followed by resuspension in 5 ml of Buffer A (10mM Hepes, pH 7.9, 10mM KCl, 1.5mM MgCl2, 0.5mM DTT) and incubation on ice for 5 min. Swelling of the cells in the hypotonic buffer was checked under a light microscope. Because nucleoli of cultured mammalian cells disassemble at 37°C in hypotonic conditions the suspension was kept at 4°C at all times (Zatsepina et al., 1997). The cell suspension was transferred to a pre-cooled Dounce homogenizer (Wheaton 0.0010" - 0.0030" clearance) and homogenized. Cell disruption was checked under the light microscope every ten strokes. Homogenization was stopped when 90% of the cells were lysed leaving intact nuclei with various amounts of cytoplasmic material attached. Next, the suspension was centrifuged at 218 g for 5 min at 4°C. The pellet contains enriched but not highly pure nuclei. The nuclei pellet was resuspended in 3 ml S1 solution (0.25M Sucrose, 10mM $MgCl_2$) and layered over 3 ml of S2 solution (0.35M Sucrose, 0,5mM $MgCl_2$). After centrifugation at 1430 g for 5 min at 4°C the nuclear pellet was resuspended in 3 ml S2 solution and sonicated for 6x 10 second bursts with 10 second intervals between each burst. Sonication was performed on ice. Sonicated nuclei were inspected under the microscope. Isolated nucleoli were identified as dense refractive bodies. The sonicated suspension was then layered over 3 ml of S3 solution (0.88M Sucrose, 0,5mM $MgCl_2$) and centrifuged at 3000 g for 10 min at 4°C.

The pellet contains nucleoli, which were further purified by resuspension of nucleoli in 0.5 ml S2 solution followed by centrifugation at 1430 g for 5 min at 4°C. The resulting nucleolar fraction was used for pull-down experiments.

Isolation of mouse ClpX

The predicted mature form of mouse ClpX (BC061153, aa 66-634) was expressed from pET28b with N-terminal fusion to His_6SUMO, by induction with 0.5 mM IPTG at 22°C for 4 h in BL21(DE3) *E. coli*. Cell pellets were lysed by French pressing (25 mM Hepes pH 8.0, 400 mM NaCl, 100 mM KCl, 2 mM $MgCl_2$, 10% glycerol, and 10 mM βME). Lysates were supplemented with 5 U/mL Benzonase, 0.5 mM PMSF, and 1/500 Calbiochem protease inhibitor cocktail 3 and cleared (30 000 x *g*, 20 min). The fusion protein was isolated by Ni^{2+}-NTA affinity (Qiagen), dialyzed overnight (25 mM Hepes pH 7.6, 300 mM KCl, 2 mM $MgCl_2$, 10% glycerol, and 10 mM βME) and cleaved with Ulp1 protease. His_6SUMO fragment and uncleaved protein was removed by incubation with Ni-NTA resin. Cleaved protein was further purified using a Bio-Gel HT HAP column (Bio-Rad), eluting with a 0-0.26M potassium phosphate (pH 7.2) gradient. Peak fractions were pooled and incubated with Q Sepharose Fast Flow resin (Amersham Pharmacia). The flowthrough, containing mouse ClpX, was concentrated, diluted tenfold in dialysis buffer (with 1 mM DTT instead of 10 mM βME), reconcentrated, and flash frozen.

References

Al-Furoukh, N., Goffart, S., Szibor, M., Wanrooij, S. and Braun, T. (2013). Binding to G-quadruplex RNA activates the mitochondrial GTPase NOA1. *Biochim Biophys Acta* **1833**, 2933-42.

Igwe, E. E., Essler, S., Al-Furoukh, N., Dehne, N. and Brüne, B. (2009). Hypoxic transcription gene profiles under the modulation of nitric oxide in nuclear run on-microarray and proteomics. *BMC Genomics* **10**.

Melchior, F. (1998). Nuclear Protein Import in a Permeabilized Cell Assay. *Methods in Molecular Biology* **88**, 265-273.

Zatsepina, O. V., Dudnic, O. A., Todorov, I. T., Thiry, M., Spring, H. and Trendelenburg, M. F. (1997). Experimental induction of prenucleolar bodies (PNBs) in interphase cells: interphase PNBs show similar characteristics as those typically observed at telophase of mitosis in untreated cells. *Chromosoma* **105**, 418-30.

Supporting Table S1

Compound Names (1.33% hits of 1280 substances)	Class	Action	Selectivity	Description	Screen 1[#]	Screen 2[##]
Flunarizine dihydrochloride	Ion Pump	Blocker	Na+/Ca2+ channel	Na+/Ca2+ channel blocker, vasodilator	+	+
5-(N,N-Dimethyl)amiloride hydrochloride	Ion Pump	Blocker	Na+/H+ Antiporter	Selective blocker of Na+/H+ Antiporter	+	+
Quazinone	Cyclic Nucleotides	Inhibitor	PDEIII	Phosphodiesterase III (PDEIII) inhibitor	+	+
Trequinsin hydrochloride	Cyclic Nucleotides	Inhibitor	PDEIII	Phosphodiesterase III (PDEIII) inhibitor	+	+
9-Amino-1,2,3,4-tetrahydroacridine hydrochloride	Cholinergic	Inhibitor	Cholinesterase	Cholinesterase inhibitor	+	-
(±)-2-Amino-4-phosphonobutyric acid	Glutamate	Antagonist	NMDA	NMDA glutamate receptor antagonist	+	-
PK 11195	GABA	Antagonist	Benzo-diazepine	Peripheral Benzodiazepine receptor antagonis	+	-
Chelerythrine chloride	Phophorylation	Inhibitor	PKC	PKC inhibitor; affects translocation of PKC from cytosol to plasma membrane	+	-
Cyclosporin A	Phophorylation	Inhibitor	Calcineurin phosphatase	Calcineurin phosphatase inhibitor; immunosuppressant	+	-
Farnesylthiosalicylic acid	G-protein	Antagonist	Ras	Non-toxic Ras inhibitor	+	-
Ethosuximide	Anticonvulsant			inhibits voltage-gated sodium channels	+	-
4-Amino-1,8-naphthalimide	Apoptosis	Inhibitor	PARP	Poly(ADP-ribose) polymerase (PARP) inhibitor	+	-
Calmidazolium chloride	Intracellular Calcium	Inhibitor	Ca2+ ATPase	Potent inhibitor of calmodulin activation of phosphodiesterase; strongly	+	-
GW2974	Phophorylation	Inhibitor	EGFR/ErbB-2	Dual EGFR and ErB-2 receptor tyrosine kinase inhibitor	+	-
GW5074	Phophorylation	Inhibitor	Raf1 kinase	cRaf1 kinase inhibitor	+	-
SQ 22536	Cyclic Nucleotides	Inhibitor	Adenylyl cyclase	Adenyly cyclase inhibitor	+	-
U0126	Phophorylation	Inhibitor	MEK1/MEK2	Specific inhibitor of MEK1 and MEK2 (MAPKK)	+	-

[#] Treatment of NOA1-EGFP overexpressing C2C12 cells
[##] Treatment of C2C12 cells and staining for endogenous NOA1

Supporting Figure S1

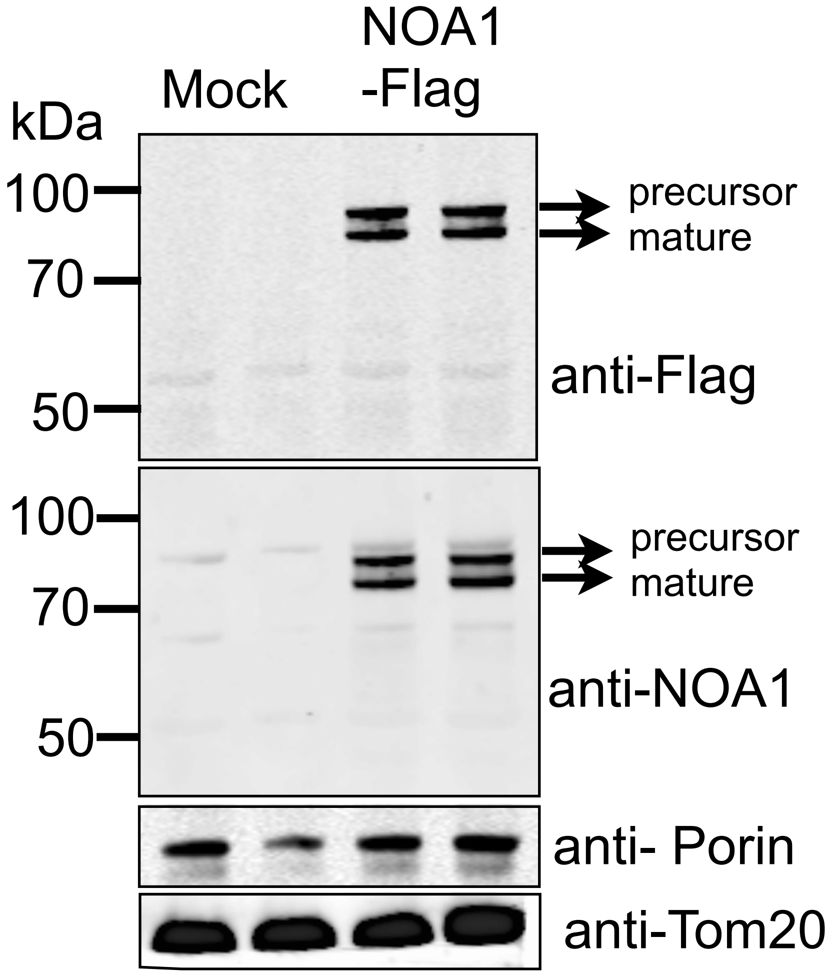

Supporting Figure S2

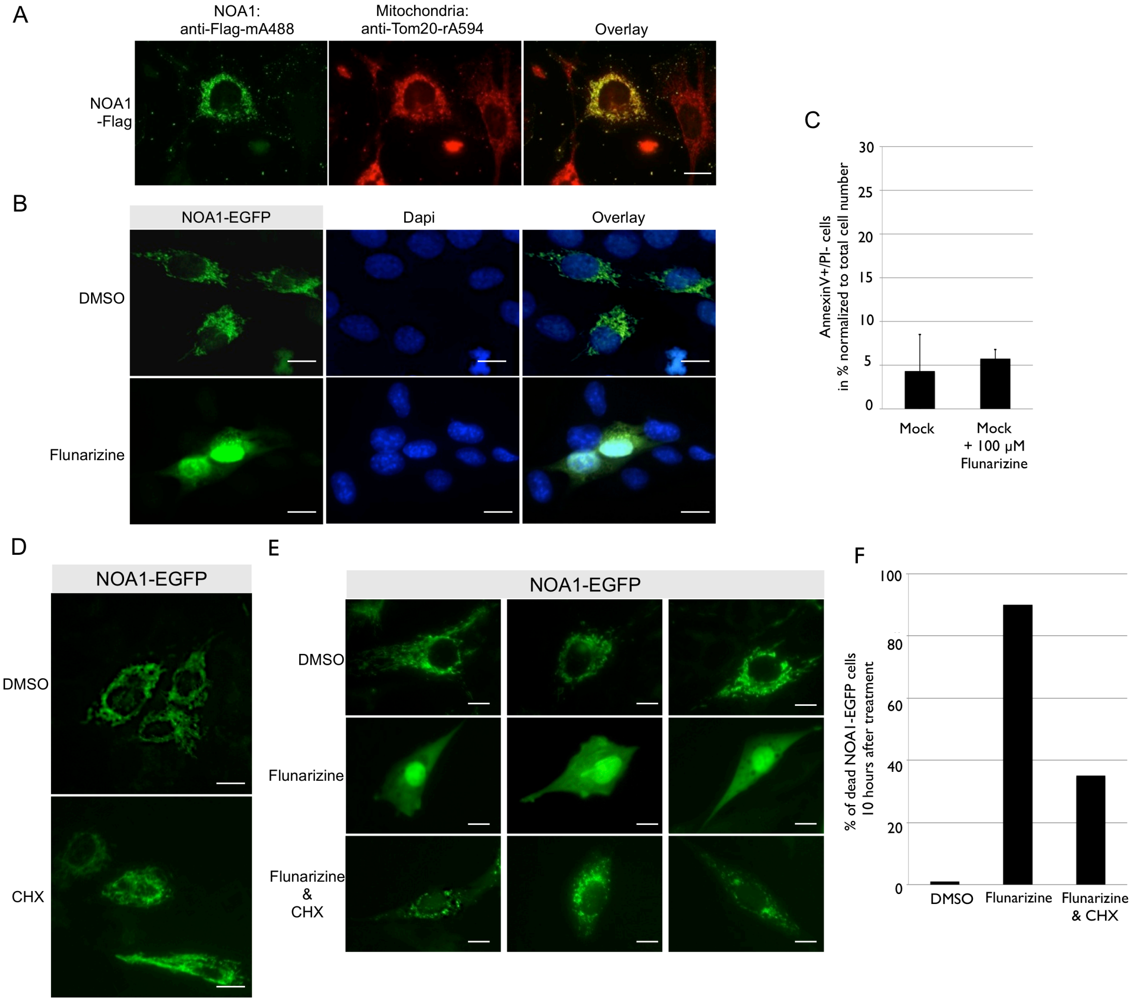

Supplement: File S1 — Supporting table and figures. Table S1, Screening of nuclear compounds initiating nuclear accumulation of NOA1. A panel of 1280 compounds (LOPAC1280 panel, Sigma Aldrich) was screened for factors causing accumulation of NOA1 in nuclei of C2C12 cells. The primary screen for compounds was evaluated by analysis of nuclear fluorescence caused by transfected NOA1-EGFP six hours after treatment with 100 µM concentrated substance in DMSO. 3.2% (41/1280) of the compounds induced increased nuclear accumulation of NOA1. 30.08% (385/1280) of the compounds induced cell death at a concentration of 100 µM. Substances causing severe toxic effects on C2C12 cells were excluded. Effects of compounds were compared to negative controls (1% DMSO and water), which did have no effects on the subcellular localization of NOA1-EGFP. All 41 compounds that were testing positive were subjected to a secondary screen based on the accumulation of endogenous NOA1 in nuclei of C2C12 cells. 17 out of 41 (1.33% of total) retested compounds consistently initiated accumulation of endogenous NOA1 in the nucleus of more than 10% of the cells. Four substances (trequinsine, quazinone, flunarizine and amiloride) were further analyzed. Flunarizine yielded the strongest effects and was therefore employed for subsequent studies. Figure S1, Rabbit polyclonal anti-NOA1 antibody is specific for precursor and mature NOA1 protein. To assess specificity of the rabbit polyclonal anti-NOA1 antibody used in this study we expressed C-terminally Flag tagged NOA1 in C2C12 myoblasts and analyzed the protein lysates by Western blotting. Flag antibody detected the larger precursor and the shorter, mature, N-terminally processed NOA1 protein. Similar result was obtained with the antibody raised against NOA1. Although highly specific, the endogenous NOA1 protein expression was below threshold for detection by Western blot. Figure S2, Flunarizine induces accumulation of NOA1 in the nucleus. (A) Immunofluorescence staining of NOA [file pone.0103141.s001.pdf]
